# Clinic Atlanta Medical College

**Published:** 1874-02

**Authors:** V. H. Taliaferro, H. F. Scott

**Affiliations:** Professor of Gynæcology; Reporter


### Clinic Atlanta Medical College.

### GYNÆCOLOGICAL.

*V. H. Taliaferro, M.D., Professor of Gynæcology.*

H. F. SCOTT, Reporter.

Case 1.—Fanny S. continued.—Feeling better; still excessive tenderness on introducing sound. Treatment: cloth tent saturated with officinal Tr. iodine; cotton and glycerine pessary. Dec. 27, cavity two and a half inches. Treatment: cloth tent saturated with carbolic acid, cotton and glycerine pessary. Constitutionally:

| | |
|---|---|
| ℞.—Potassii Bromid . . . . . . . | gr. v. |
| Tr. Ergotæ . . . . . . . . | ʒ. ss. |

M. Ft. Haust. Sig. To be repeated half-hour before each meal. Jan. 10, tumor much reduced, local and general treatment continued.

Case 2.—Mrs. Carrie B. continued.—Dec. 20. Same displacement; cavity same; no ulceration. Treatment: cloth tent coated with iodine ointment; cotton and glycerine pessary. Jan. 17. Uterus looks healthy; little leucorrhœa; cavity same. Treatment: Churchill's caustic iodine applied to vagina and cervix with camel-hair brush.

Case 3.—Julia A. continued.—Dec. 13. Treatment: solid tent nitrate silver; cotton and glycerine pessary. Jan. 3. Decided improvement. Treatment continued with application of Tr. iodine to vagina. January 10. Ulceration disappeared; cavity two and a half inches. Treatment: cloth tent saturated with Churchill's caustic iodine, and coated with iodine ointment; Tr. iodine to vaginal wall; cotton and glycerine pessary. January 17. Regular improvement. Treatment continued. Constitutionally:

| | |
|---|---|
| ℞.—Potassii Iodid . . . . . . . | gr. v. |
| Extract Ergot, Fluid . . . . . . | ʒ ss. |
| Aquæ . . . . . . . . . . | ℥j. |

M. Ft. Haust. Sig. To be repeated half-hour before each meal. January 24. Local and general treatment continued.

CASE. 4.—Martha S. continued.—January 24. Leucorrhœa not so profuse; plug of tenacious mucus in cervical canal; sound causes considerable pain; cavity same. Treatment: cloth tent coated with iodine ointment; cotton and glycerine pessary.

CASE 6.—September 13. Jane B., æt. thirty-two, colored, washerwoman, married fourteen years, four children, aged eighteen to nine respectively; no abortions. Medium size, muscular, very restless and sleeps very poorly; pain in head, bowels and back, especially marked about menstrual period. Menstruated at fifteen; now regular. *Vaginal touch,* cervix enlarged, quite hard, granular, occupying its normal position; entire uterus enlarged and sensitive. *Speculum:* Cervix and os much congested, abrasion of epithelium around the os. Sound gives some pain, meets with slight obstruction at internal os; passes upward in normal direction; slight flow of blood on withdrawal; cavity three inches. Diagnosis: Subinvolution and general endometritis. Treatment: Cloth tent saturated with carbolic acid, and coated with strong ointment of iodine; cotton and glycerine pessary. November 1. Depth same. Treatment: Cotton wrapped probe, dipped in carbolic acid, introduced. November 29. Had an acute attack of pleurisy since was here last; uterus bleeds on slightest touch; large and congested; depth three and one-quarter. Treatment: Solid tent nitrate silver introduced. December 27. Ulceration almost disappeared. Treatment: Tent of carbolic acid introduced; cotton and glycerine dressing. Constitutionally:

| | |
|---|---|
| ℞.—Potassii Iodid . . . . . | gr. v. |
| Extract Ergot, Fluid . . . | gtts. xx. |
| Aquæ . . . . . . | ℥ j. |

M. Ft. Haust. Sig. To be repeated half an hour before each meal. January 10. Condition about same. Local and general treatment continued, with addition of iodine ointment to tent. January 24. Local and general treatment continued.

CASE 7.—December 26. Amy A., æt. twenty-three, colored, laundress; menstruated about sixteen; married seven years; no children; abortion at five months, two years ago. Suffers from metrorrhagia, pain in hypogastrium, hips and back. No examination made, on account of metrorrhagia. Constitutionally:

| | |
|---|---|
| ℞.—Potassii Bromid . . . . . . | gr. v. |
| Extract Ergot, Fluid . . . . . | gtts. xx. |
| Aquæ . . . . . . . . . | ℥ j. |

M. Ft. Haust. Sig. Repeated half an hour before each meal. January 3. Metrorrhagia ceased. For constipation:

℞.—Aloes Extracti,
Ferri Sulphat. . . . . . aa. gr. xxiv.
Belladonnæ . . . . . . gr. iv.

M. Ft. Pill. No. twenty-four. Sig. One, three times daily. January 10. Vaginal touch: Cervix indurated and too high in pelvis. Bimanual palpation: Fail to bring uterus between the fingers. Rectal touch: Suspect fibroid tumor. Speculum: Cervix very large. Sound: Reveals a fibroid tumor, size of a small orange; depth three and one-quarter. Treatment: Tent coated with iodine ointment; cotton and glycerine pessary, applied tri-weekly; constitutional treatment continued.

Case 8.—October 4. Mary E., æt. eighteen, colored, house servant; menstruated at thirteen; unmarried; no conception; complains for one year of pelvic pains and absence of menstruation; medium size; nutrition good; well developed muscular system. Taxis: Vaginal walls hot and intensely red; cervix very small, button-shaped, directed towards vaginal outlet; fundus towards hollow of sacrum. Speculum: Profuse muco-purulent discharge. Sound: Passes upwards and backwards; cavity of uterus two and one-quarter inches. Diagnosis: Retroflexion and specific endometritis. Treatment: Cloth tent saturated with carbolic acid; cotton and glycerine pessary; os painted with Tr. iodine. October 11. Feeling better. Treatment continued. October 18. Still improving. Treatment continued. October 25. Condition unchanged. Treatment continued. November 1. Very little leucorrhœa now; general appearance of cervix and mucous membrane improved. Treatment: Tr. iodine to vaginal cervix; muriate of ammonia twenty grs. three times daily. Nov. 29. Improving; no treatment; muriate ammonia continued. December 20. Feels very well, with exception of vertigo once or twice daily; still slight endo-metritis; depth same. Treatment continued.

Case 9.—November 22. Amanda Patrick, æt. nineteen, colored, unmarried, no children, no miscarriages. This patient was treated during vacation. Had general endometritis extending into *cul de sacs;* granular ulceration of entire os and *cul de sacs.* Now almost well; no ulceration; uterus looks nearly well; probe cannot be passed within internal os. *Treatment:* Cloth tent coated with iodine ointment introduced to internal os; cotton pessary. November 29th. Very sick after last treatment; confined to bed last menstruation; globus hystericus, especially during menstruation; uterus a little tender; slight leucorrhœa. *Treatment:* Solid tent nitrate silver, cotton pessary. December 13th. Rheumatism in shoulder and intercostal muscles.

*Treatment:* Potassii Iodid, . . . . . gr. v.
Tr. Colchici, . . . . . . gtts. xv.

Three times daily. December 20th. Micturates with difficulty; some ulceration, probably result of nitrate of silver; depth, two and a half inches. Treatment: Cantharidal collodion to cervix, cotton pessary. December 27th. Condition same. Treatment: Cloth tent saturated with carbolic acid and coated with iodine ointment, cotton and glycerine pessary. January 3d, 10th and 17th. Treatment continued. January 24th. Improved. Treatment continued.

Case 10.—December 6th. Emeline B., æt. twenty-eight, colored, cook. Menstruated at fifteen; no children; aborted at six months, five years ago; health bad for five years; complains of pain in hypogastrium, loins and back; suppression of menstruation for three years. Examination: cervix small and too high in pelvis, being nearly as high as fundus is normally. *Bimanual taxis:* cannot bring uterus between hands. *Rectal touch:* little round mass back against rectum, firmly fixed in its position. Sims' Speculum: very minute os; ulceration of os and cervix; profuse yellow leucorrhœa. Sound: cannot be introduced either in this or ordinary position. Knee-elbow position: Mass is not moved by changein position; find that the mass is the cervix which is now well displayed; sound introduced through very minute opening, passes anteriorly; depth, two and one-quarter inches. *Diagnosis:* Atrophy, the result of superinvolution. Treatment: cloth tent for dilatation. December 20th. Condition about the same. Treatment: cloth tent saturated with carbolic acid and coated with iodine ointment, cotton and glycerine pessary. December 27th. Some improvement; uterus pliable and easily brought between hands; probe and tent easily introduced; depth of cavity, two and three-eighths inches. Treatment continued. January 1st. Marked improvement; uterus is fast resuming its normal position; uterine cavity two and seven-eighths inches. Treatment continued. January 10th. Still improving; some ulceration of anterior lip; depth, three and one-eighth inches. Treatment continued.

Case 11.—November 22d. Mary T., æt, twenty-four, colored, seamstress. Menstruated at fifteen, three children, aged five years to seven months, respectively. No abortion; complains for seven months of pain in head, back and hips; tenderness of dorsal vertebræ; appetite good; bowels regular; menses regular every twenty-eight days, continuing four days; confined to bed often during the whole period. Examination: cervix enlarged, congested and patulous; too low in pelvis; abdomen somewhat tender. *Sound:* passes in normal direction; cavity, three and one-quarter inches. Diagnosis: Sub-involution. Treatment, none. November 29. Cavity, three and three-eighths inches. Treatment: solid tent nitrate of silver, cotton and glycerine pessary. December 20th. Sick for one week since last treatment; suffered considerably; cavity three and one-eighth inches. Treatment: cloth tent saturated with carbolic acid and coated with iodine ointment, cotton and glycerine pessary. January 24th. Cervix still large; cavity three and one-eighth inches. Treatment: cloth tent coated with iodine ointment, cotton and glycerine pessary. Constitutionally:

| | |
|---|---|
| ℞.—Potassii Iodid. . . . . . . | gr. v. |
| Extract Ergot, Fluid. . . . . . . | ʒ ss. |
| Aquæ, . . . . . . . . | ℥ j. |

M. Ft. Haust. Sig. To be repeated half an hour before each meal.

